# Modified Small-Volume Jet Nebulizer Based on CFD Simulation and Its Clinical Outcomes in Small Asthmatic Children

**DOI:** 10.1155/2019/2524583

**Published:** 2019-06-10

**Authors:** Sermsri Santati, Jatuporn Thongsri, Parinya Sarntima

**Affiliations:** ^1^Ramathibodi School of Nursing, Faculty of Medicine Ramathibodi Hospital, Mahidol University, Bangkok 10400, Thailand; ^2^Computer Simulation in Engineering Research Group, College of Advanced Manufacturing Innovation, King Mongkut's Institute of Technology Ladkrabang, Bangkok 10520, Thailand

## Abstract

The small-volume jet nebulizer (SVJN) is an aerosol device used to treat respiratory illnesses. Major problems for aerosol treatment in small children include the penetration of particles to the lower lungs due to irregular and small volume of a child patient's breath while the nebulizers used are the same models intended for adults. This adult SVJN produces a huge number of particles at a higher speed than small children can intake. To solve this problem, computational fluid dynamics (CFD) was used to redesign the device by adding 6-inch corrugated tube with 80 ml capacity (equal to one inhale capacity of a small child) into the traditional SVJN. Results revealed that the undulations of the corrugated tube were the important parts that change the direction of aerosol flow, slowing down the produced speed of aerosol up to 31.48% (mean speed = 0.37 m/s via modified SVJN vs 0.54 m/s via traditional which were close to measured results). The modified SVJN was tested for the effectiveness on how it could accommodate bronchodilator drug to the lower lungs by 3 clinical researches with 238 asthmatic children aged 1–5 years. The results revealed that the experimental group reported higher bronchodilatating effects: higher mean score of change in oxygen saturation and degree of wheezing and greater reduction in respiratory rate per minute than the control group with statistical difference (*p* < 0.05). Meanwhile, heart rate and physical attributes (dead volume and duration of aerosol treatment) were indifferent. Moreover, small children showed more acceptance behavior towards this modified SVJN than the traditional one. Modified SVJN might be a good choice for aerosol treatment in small children because it slows down the speed of aerosol production, makes them well spread all over the reservoir, and is ready for small children to inhale for better clinical outcomes while physical attributes are the same.

## 1. Introduction

Aerosol therapy is used to treat patients with respiratory problems using drug mists [1]. This treatment delivers respiratory drugs directly to the lower lungs. The method is safe and more effective with less side effects compared to intravenous and oral medication [2, 3]. However, aerosol therapy in small children is considered problematic as the drug percentage absorbed into the young patient's lungs is lower than in grown children and adults. The problem is caused by children's smaller respiratory tracts, which can naturally contain less tidal volume and respiratory rate. Also, the present device and medication terms were designed for adults, not for small children. When used on children, most of the drug is lost into the surroundings while only a small amount can enter the patient's lower lungs. The worse scenario is that if a child cries or has other resisting reactions during treatment, the amount entering his/her lower lungs will be even lower, negatively affecting treatment [3]. Therefore, adjusting the nebulization device to suit small children is necessary for improving medical results.

From the literature review, we learned that most modified aerosol devices were done only in laboratories, yet practical usage of the adjusted device on children remain low because clinical practice requires safety approval from the Hospital's Board of Research Ethics, which is time-consuming and has many procedures. Samples of related research include Amirav et al.'s work [4, 5] who reported about using aerosol treatment in bronchiolitis patients using a hood nebulizer along with an aerosol face mask, which improved the average lung deposition and clinical results when compared to using the aerosol face mask alone. Thus, the hood nebulizer is an example of successful case of device adjustment that improves treatment efficiency once added to aerosol therapy. Still, using the hood nebulizer may not be the best solution for treating small children since the hood may scare or cause negative reactions that will interrupt therapy. Most importantly, Amirav et al.[4, 5] did not compose a detailed report on the terms and conditions related to using the hood nebulizer, which may be inconvenient if practiced in a real situation. When devices are modified for a real clinical use, it is a vital for all researchers to be aware of the patients' safety including working principles, related theories, and suitable terms and conditions, along with any limitations of the adjusted device. To better understand these aspects, we must integrate engineering and nursing knowledge together. Therefore, this research is a collaboration between researchers who expertise in related fields aiming to modify the actual small-volume jet nebulizer used in small children effectively and knowledgeably, with both medical principles and theories.

As for the engineering field, small-volume jet nebulizer (SVJN) adjustment requires computational fluid dynamics (CFD) simulation. By setting proper boundary conditions according to actual nebulization, the CFD will simulate and display the velocity vectors and pathlines of drug particles within the SVJN. The results can later be analyzed to find the most suitable condition for treating small children, the concept of modification for actual SVJN usage with higher efficiency. Research examples which used the CFD in a similar way to this research are by Inthavong et al.'s work [6]. They used the CFD to simulate the volume of drug particle quantity collected in the human nasal cavity to determine the best condition for designing a nasal spray device. Thongsri et al. [7–9] used the CFD to simulate airflow and contaminated particle pathlines in various industrial machines at factories to find the best condition in decontaminating particles from manufacturing. Kaewbumrung et al. [10] used the CFD to simulate drug particle flow in the blood system. They reported that the quantity of drugs affected both pressure and wall shear stress. The results of this research are used to determine the best conditions to produce medicine and treatment for blood system illnesses. Zhou et al. [11] used the CFD to analyze hemodynamic characteristics in stenosed arterial models. The results were analyzed for artery stenosis disease treatment. All mentioned researches from [6–11] are problems that occur in a steady state; contrastingly, aerosol treatment and SVJN adjustment in this research are problems that occur according to time or transient state. Many factors such as the child patient's inhale-exhale rates and profiles of fluid inside SVJN depend on time. Therefore, using the CFD in this research is more challenging and complicated than the aforementioned studies. Another difficulty is once the SVJN has been modified, nursing knowledge is required to actually clinically test the modified device. In this test, a sample group, a control group, experimental design, and suitable statistical analysis of the result must be suitably chosen to confirm usage efficiency. Once this is confirmed, the device can then be confidently used for actual therapy in hospitals. No researches have used the CFD to adjust the SVJN for enhancing aerosol therapy efficiency, including clinically using the results for small asthmatic children before.

Therefore, in this article, we shall propose the methodology of successfully using the CFD to modify and optimize the SVJN device. Clinical results of SVJN actual usage are effective in treating small child patients. The materials used to modify the SVJN in this research are easy to find and inexpensive and do not require complex procedures. The authors fully believe that this research will provide readers with knowledge for further application to improve respiratory illness therapy in the future.

## 2. Theoretical Background

### 2.1. Small-Volume Jet Nebulizer (SVJN)

**Figure 1:**
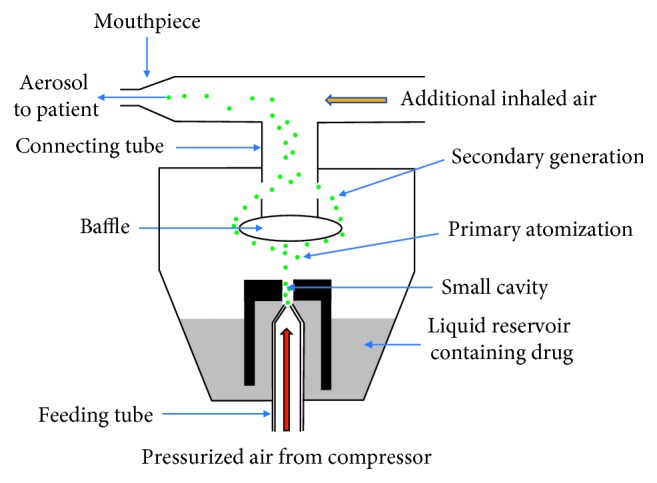
Working principle of the jet nebulizer.

This device reforms mixture of liquid formulations and drug suspension into mist of small particles. It is inexpensive and generally used in hospitals. SVJN's working principles may be concluded in Figure 1. When pressurized air (such as oxygen or high-pressure air) is supplied into the feeding tube through a small cavity, the pressure changes rapidly turning liquid and drug into mist of particles known as the Venturi effect. Drug particles produced in the beginning phase have sizes of approximately 15–500 *µ*m, so called the primary atomization. 99% of this will flow and collide with the baffle, shrinking the drug particles to a size of approximately 2–10 *µ*m, so called the secondary generation. These drug particles flow along the connecting tube and combine with additional inhaled air before entering the patient's respiratory system. Drug particles that cling to the nebulizer wall will recollect into the reservoir, ready to go through the procedure again [12, 13]. Factors that affect the size and quantity of produced drug particles are as follows: the diameter of the small cavity, pressurized airflow rate, and baffle's size [14]. At present, the SVJN may appear differently, depending on the manufacturer's design yet the working principles remain the same.

### 2.2. Governing Equation for CFD

The velocity vector of fluid within the SVJN can be calculated by solving conservation and turbulence equations. Ansys Fluent 17.1 [15], a CFD program that calculates conservation equation, consists of 3 equations; conservation of mass (1), momentum (2), and energy (3) may be presented as follows:

(1) $$\begin{array}{c} \frac{\partial \rho }{\partial t}+\frac{\partial \left(\rho u_{i}\right)}{\partial x_{j}}=0, \end{array}$$

(2) $$\begin{array}{c} \frac{\partial \left(\rho u_{i}\right)}{\partial t}+\frac{\partial \left(\rho u_{i}u_{i}\right)}{\partial x_{j}}=-\frac{\partial P}{\partial x_{i}}+F_{i}+\frac{\partial \left[\tau_{ij}\right]}{\partial x_{j}}+\frac{\partial \left(-\rho \bar{u_{i}^{\prime }u_{j}^{\prime }}\right)}{\partial x_{j}}+\frac{\partial \left(-\rho \bar{{u_{i}^{\prime }}^{2}}\right)}{\partial x_{i}}+S_{m}, \end{array}$$

(3) $$\begin{array}{c} \frac{\partial \left(\rho E\right)}{\partial t}+\frac{\partial \left[u_{i}\left(\rho E+P\right)\right]}{\partial x_{i}}=\frac{\partial \left({\left(k\right)}_{eff}\left(\partial T/\partial x_{j}\right)+u_{i}{\left(\tau_{ij}\right)}_{eff}\right)}{\partial x_{j}}+S_{h}. \end{array}$$

The turbulence model for this research is shear stress transport *k-ω* (SST *k-ω*) [16], which consists of 2 equations. SST *k-ω* is accepted for its accuracy, since it is a combination between the *k-ω* turbulence model appropriate for the inner region of the boundary layer and the *k-ε* turbulence model, which is appropriate for free shear flow [15, 16]. Therefore, the turbulence model is suitable for this research.

The particle pathlines of this drug can be calculated from the particle force balance equation in the discrete phase model (DPM) [17] represented as follows:

(4) $$\begin{array}{c} \frac{du_{p}}{dt}=F_{D}\left(u_{f}-u_{p}\right)+\frac{g\left(\rho_{p}-\rho_{f}\right)}{\rho_{p}}+F_{s}. \end{array}$$

Since the drug particle is micron-sized and its direction can change with the influence of fluid flow, *F*_*s*_ in (4) is considered to be influenced by Saffman's lift force, virtual mass force, and pressure gradient force. Saffman's lift force is an important external force towards drug particle pathlines and can be presented as follows [18]:

(5) $$\begin{array}{c} \vec{F}=\frac{2K\rho_{f}\nu^{1/2}d_{ij}\left({\vec{u}}_{f}-{\vec{u}}_{p}\right)}{\rho_{p}d_{p}{\left(d_{lk}d_{kl}\right)}^{1/4}}, \end{array}$$

where *K* is 2.594, *d*_*ij*_, *d*_*lk*_, and *d_kl_* are the deformation tensors. The direction of drug particle pathlines can change from contact with each other, or the change according to the fluid flow, or having an interaction with the continuous phase. Mentioned drug particle behaviors can be calculated using the Ansys Fluent 17.1 program. Fluent is a CFD program that calculates using the finite volume method principle. The model is divided into elements called the control volume. Physics equations inside the control volume must be conservative. When the boundary condition has been completely determined, fluent will solve all of (1)–(5) and 2 equations for the turbulence model to calculate the unknown variables in those elements as a numerical result, before displaying the results in different color shades for convenient result analysis. We shall explain the determination of necessary variables and fluent settings in the program in Section 3.3.

Afterwards we will discuss the methodology divided into 2 steps: CFD methodology and clinical methodology.

## 3. Computational Fluid Dynamics

In this chapter, we shall discuss research procedures and concepts along with CFD usage methods for successful SVJN modification.

### 3.1. Concept of Modification

**Figure 2:**
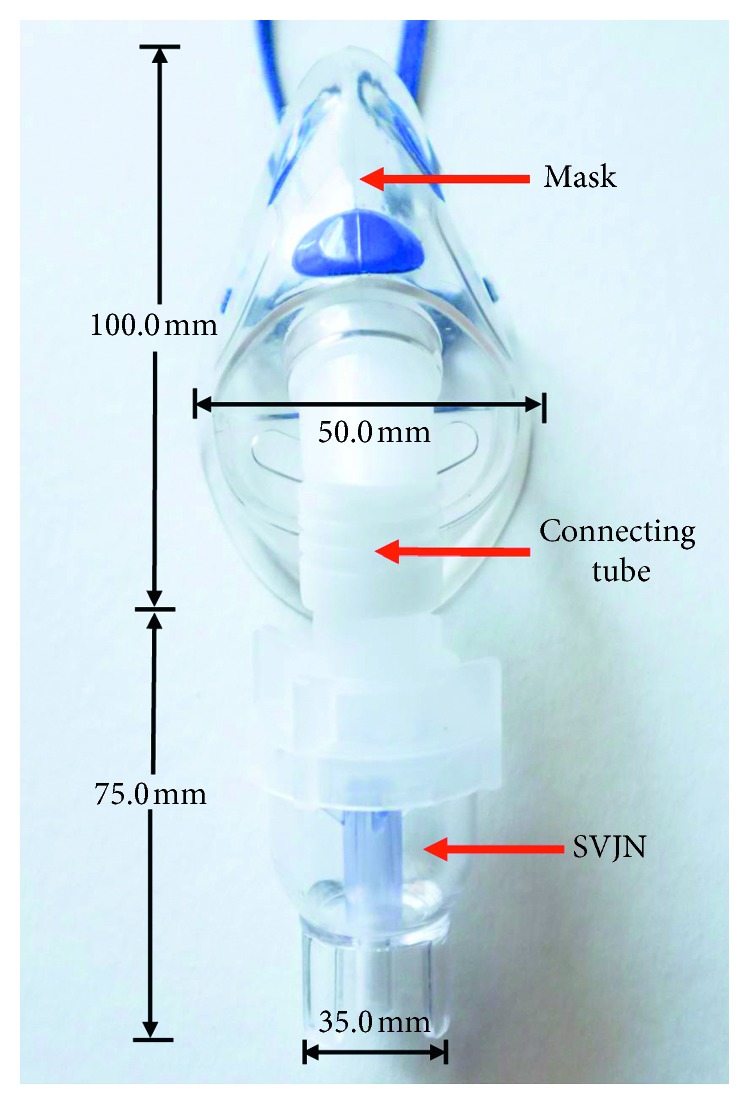
An actual SVJN device and dimension.

In this section, we shall explain the modification guidelines.  Figure 2 shows an actual SVJN device and dimension consisting of the SVJN, mask, and connecting tube. This SVJN is the model actually used in the hospital, which appears slightly different from the model mentioned in Section 2.1, as designed by the manufacturer. Still, the working principles remain the same. The connecting tube is the part that connects the SVJN and mask together. This is optional depending on the user. The lower part of the SVJN has a channel connected to the air duct that passes from the compressor. The upper part has a duct connected to the mask. The mask type we use has a hole opening at the bottom, near the patient's mouth. In the past, the aerosol devices had problems and were not fully effective because of the device's design, or sometimes the child patients cry or show resisting behavior, lessening the quantity of drug particles entering their system. To resolve this problem, we aim to modify the SVJN by adding 6-inch corrugated tube with 80 ml capacity into the traditional SVJN.

### 3.2. Fluid Model and Mesh Model

**Figure 3:**
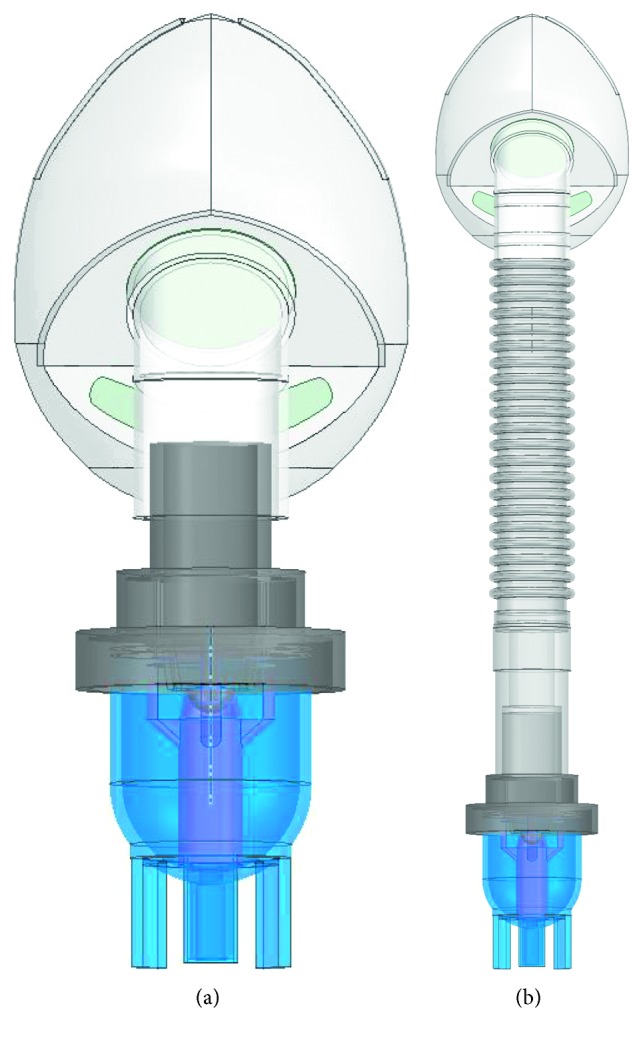
Solid models of (a) traditional and (b) modified SVJNs.

**Figure 4:**
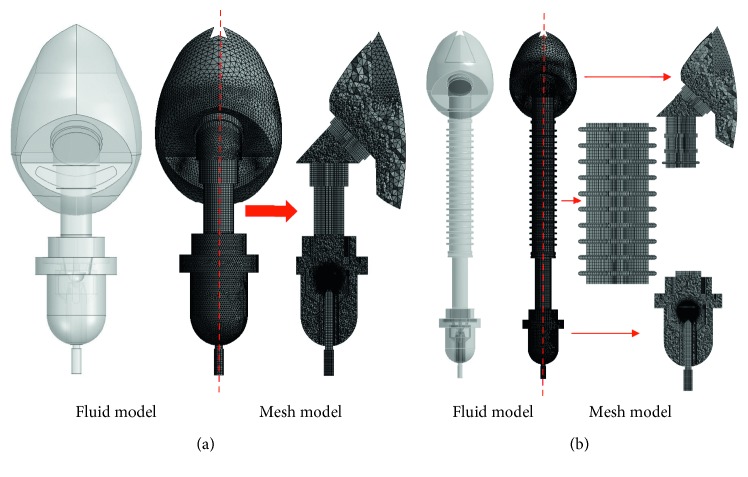
Fluid and mesh models of (a) traditional and (b) modified SVJNs.

We used the model in Figure 2 as the prototype to create solid models shown in Figure 3 for the traditional (Figure 3(a)) and modified (Figure 3(b)) SVJNs.  Figure 4 shows the fluid and mesh models of the traditional (Figure 4(a)) and the modified (Figure 4(b)) SVJNs. The newly modified mesh model is hybrid, consisting of both hexahedrons and tetrahedrons which give a lower skewness, lesser than 0.85. From the mesh-independent analysis, we found that the mesh models are suitable in both qualities, can provide accurate answers, and uses appropriate calculation time of 594,725 nodes and 3,228,243 elements for the traditional SVJN and 816,448 nodes and 3,396,049 million elements for the modified SVJN. To set the inhale-exhale conditions of child patients, we set the diameter of a child's nostrils to 6 mm at a realistic position within the mask.

### 3.3. Fluent Settings

Boundary conditions are set to be most similar to the actual situation. We then calculate the fluid's velocity vector and drug particle pathlines of the traditional SVJN compared to the modified SVJN when the respiratory rate is 30 and 40 time/min and the oxygen flow rate is 6 and 8 L/min. Therefore, 8 cases must be simulated.  Table 1 concludes research conditions.

**Figure 5:**
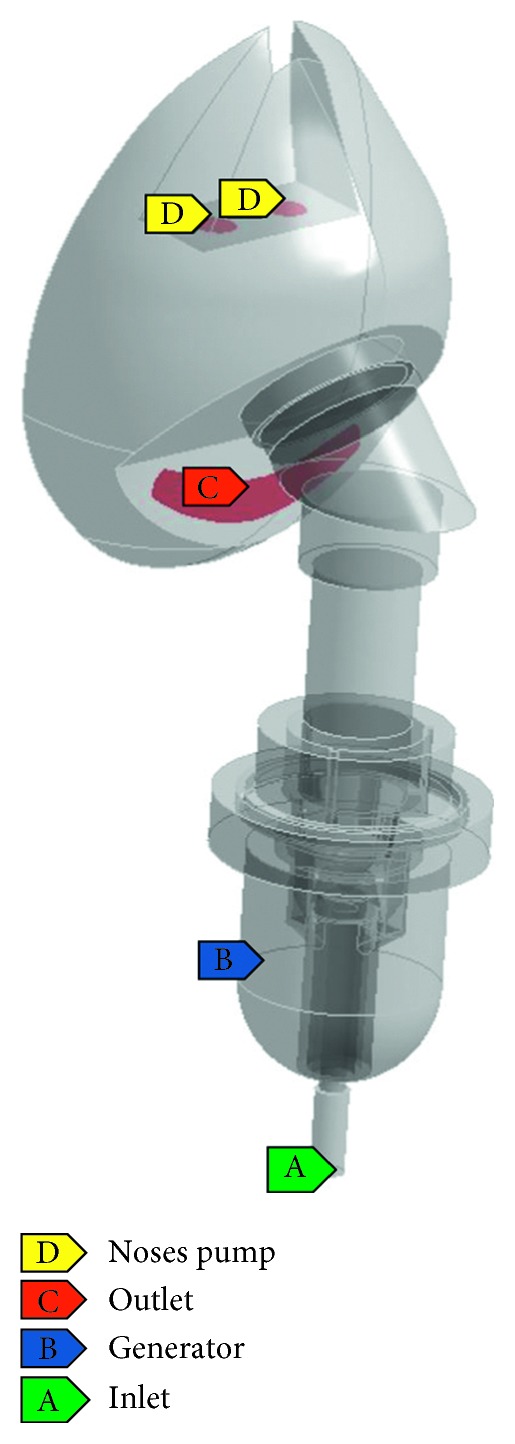
Positions and types of boundary conditions.

These factors were set in fluent settings. We set the fed fluid as oxygen. Both oxygen and drug particles can exit the system only at 2 channels; the nostrils and opening of the mask's hole. Since we do not know the drug and exhaled air's material properties and exhaled air has a small volume compared to the volume of fed oxygen, we assumed the drug particles to be spherical as in oxygen with diameter of 2 microns, and density of 1.299 kg/m^3^. The pressurized air was oxygen gas fed into the device with a density of 1.331 kg/m^3^. We set the respiratory rate to be 30 and 40 times per minute, the normal breathing rate of small children. Respiratory rate is the number of breaths you take per minute (time/min). The normal respiratory rate for an adult at rest is 12 to 20 time/min but for a child it is 30 to 40 time/min. The child's lung capacity is set at 80 cc, which is equal to one inhale capacity, or tidal volume in small children. The turbulence model used is shear stress transport (SST) *k-ω*. The DPM is set as unsteady particle tracking and interaction with the continuous phase. The boundary condition at wall was set to reflect. The forces towards drug particles are Saffman's lift force, gradient force, and virtual mass force. The solver used is pressure-based in transient state. Solution methods are set to coupled. Spatial discretization for pressure, momentum, turbulent kinetic energy, and specific dissipation rate is set to the second order which is the highest quality of the program. The bottom inlet has an oxygen flow rate of 6 or 8 L/min. This flow rate makes drug particles suitable for asthmatic treatment [2]. We set the area around the opening mask's hole with pressure gauge = 0 Pa, which is the outlet. The nostrils' role is like a pump that suction oxygen out from the mask when the child inhales and pumps oxygen into the mask when the child exhales. As for the nose pump, we set the user-defined function (UDF) to control the inhale-exhale rate at the nostrils. The oxygen quantity of 1 inhale or exhale equals 80 cc. The simulation to find particle pathlines uses the DPM and the same technique that made [7–9] successful. The reservoir acts like a generator that releases drug particles according to the preset time. We could not simulate drug particles released at all times due to computer performance limitations. We simulated by releasing them 6 times at 0.000 s, 0.005 s, and 0.010 s (inhaling phase), 0.800 s, 0.805 s, and 0.810 s (exhaling phase), 2,400 particles per time, totaling 14,400 particles to release. We simulated during the first 5 seconds, the crucial time that affects changes in results. We used 0.005 s of time step size, 1,000 time steps, and 15 iterations per time step. Thus, the computer must calculate a total of 15,000 iterations per case to show the simulated results for 5 s. We were positive that all the values set for the simulation are realistic and can calculate the velocity vectors, drug particle pathlines within the SVJN, and summarize the modified model's performance.  Figure 5 shows positions and types of the boundary conditions. In Chapter 5, we shall present the simulation results and discussion and result accuracy verification and compare performance analysis of the traditional and modified SVJNs. The modified SVJN will be clinically tested and will be discussed in the following section.

## 4. Clinical Test

Processes in testing clinical outcomes were done by 3 clinical research studies. All of the 3 studies used a quasi-experimental, pretest-posttest design to investigate the following: (1) the effectiveness of the modified model on how small children accept this redesigned equipment; (2) how the equipment's work by measuring 2 physical attributes (dead volume-volume that remains in the equipment after each treatment and duration of aerosol treatment); and (3) how it could promote the bronchodilator effect by measuring 4 clinical outcomes (oxygen saturation, respiratory rate, heart rate, and degree of wheezing) compared with the traditional one. A total of 238 children aged 1–5 years who were diagnosed by physician as having bronchospasm were recruited. The experimental group received an aerosolized bronchodilator (Salbutamol) by using the modified device while the control group used the traditional adult sized one. Before and after 15 minutes of each aerosolized bronchodilator, the researcher assessed the acceptance behavior, physical attributes, and clinical outcomes. The difference changes of the outcomes between control and experimental group were analyzed and tested for the significant differences by using the Mann–Whitney *U* test and ANCOVA.

## 5. Results and Discussion

### 5.1. CFD Simulation

**Figure 6:**
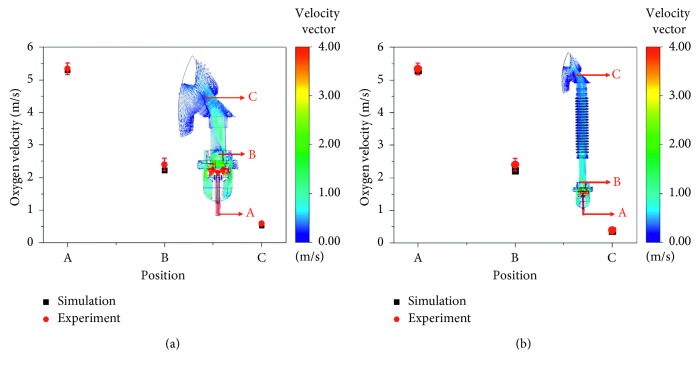
Comparison between the measured and the simulated oxygen velocity for (a) traditional and (b) modified SVJNs at positions A, B, and C.

In order to confirm the simulation results using the CFD, we experimented by measuring the oxygen velocity fed into the SVJN during the time when no breathing had occurred. Both models were measured at positions A, B, and C for 20 times before finding an average value. The oxygen flow rate was 8 L/min supplied from an actual compressor of the hospital. We measured the velocity with a hot-wire anemometer with an accuracy of ±0.03 m/s.  Figure 6 compares the oxygen velocity results which were actually measured with the results calculated from simulation at positions A, B, and C for 6(a) traditional and 6(b) modified SVJNs.  Table 2 shows the results of comparison.

Notice that in Table 2, the oxygen velocity at positions A, B, and C obtained from measurement and simulation from the traditional and modified SVJNs is consistent. The standard deviation (SD) is lesser than 0.19 m/s, and the error value is lesser than 8.71%. The error value might be because we used a single value of oxygen flow rate for simulation, but the actual one from experiments is unsteady flow. For example, we used an average of 8 L/min at inlet for simulation, but, in fact, it was in a range of 7.8–8.2 L/min for the measurement. The oxygen velocities at positions A and B of both models are less different than at position C because they were from the starting phase of oxygen flow, so adding a corrugated tube does not affect the velocity at these areas. At position C, the simulation of oxygen velocity of the modified SVJN equaled 0.37 m/s, lesser than conventional one which was 0.54 m/s. This indicates that the corrugated tube helps slow oxygen flow before entering the mask by 31.48%. We will not report the 6 L/min oxygen flow in this article, but the compared results are consistent. Thus, the selected methodology and simulation are both accurate and creditable.

**Figure 7:**
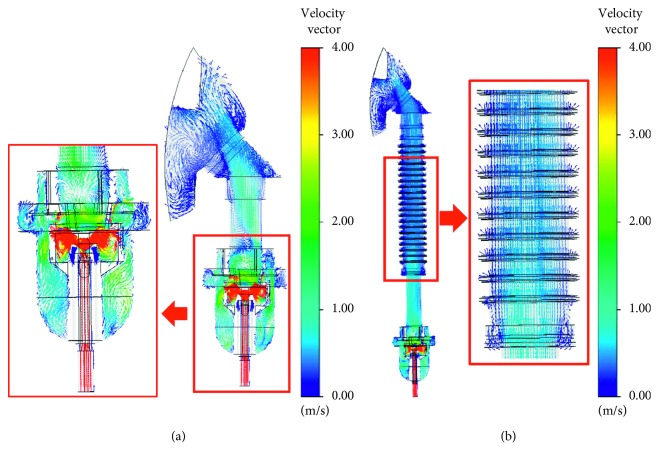
Oxygen velocity vectors for (a) traditional and (b) modified SVJNs.

To investigate oxygen flow behavior, Figure 7 shows oxygen velocity vector within 7(a) traditional and 7(b) modified SVJNs with 8 L/min of oxygen flow rate at a steady state. The red areas are where velocity exceeds 4 m/s. Before entering the small cavity, oxygen velocity is approximately 4.14–4.67 m/s. However, when passed through the small cavity, the velocity increases by over 100 times, or between 476 and 579 m/s. This velocity is consistent with the numerical and experimental results reported by Lelong et al. [19], a study of the atomization process in a jet nebulizer. According to the Venturi effect principle, the higher the velocity of a fluid, the lower the pressure. Oppositely, the lower the velocity, the higher the pressure. Many small drug particles occur in this area consistent to the theory mentioned in Chapter 2.1. For a greater understanding, readers should consider Figure 1 alongside. Afterwards, the tiny drug particles will float within the reservoir before moving towards the connecting tube and eventually the mask. Here, the velocity reduces to approximately 0.59 m/s in the traditional SVJN, waiting for the child patient to inhale. Comparing Figures 7(a) to 7(b), notice that the length and indents of the corrugated tube reduce the velocity more than the model without one. Position C in the modified SVJN lessens oxygen velocity to 0.39 m/s in the experiment.  Figure 7(b) also shows that the corrugated tube's indents causes turbulence, that helps keep drug particles in the SVJN for longer periods.

**Figure 8:**
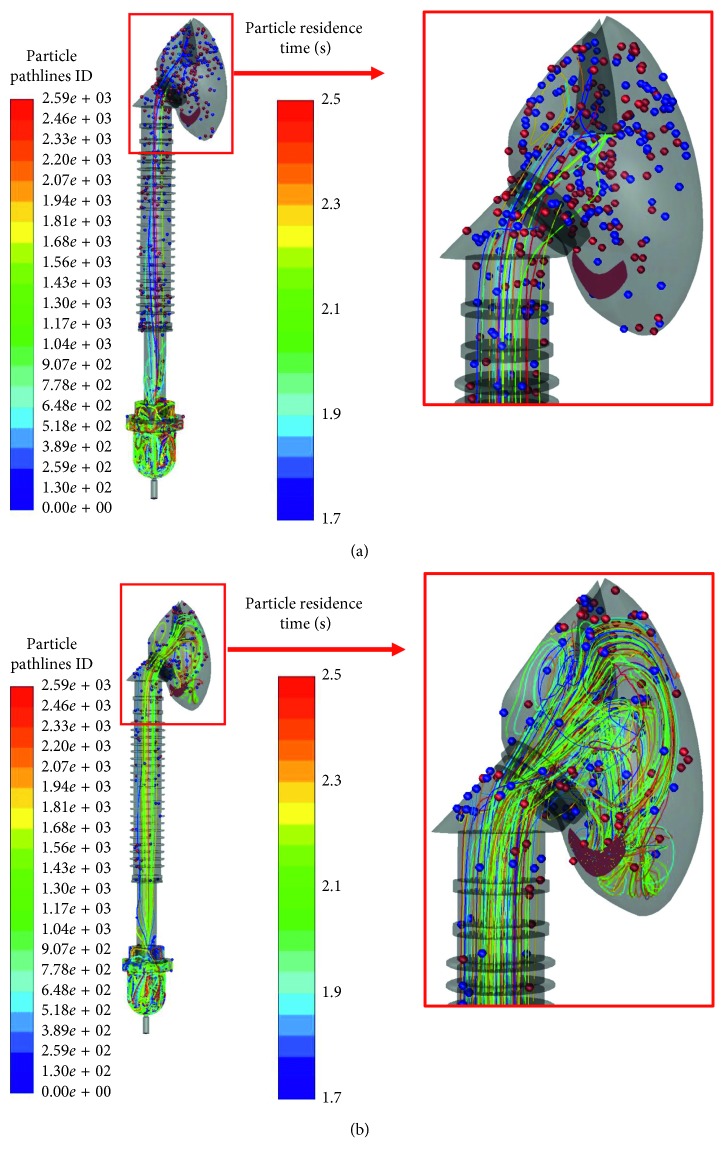
Drug particle pathlines for after (a) 2.5 s and (b) 3.5 s from starting time.

To examine drug particle pathlines, in our simulation, we released 14,400 oxygen particles at the reservoir as explained in Section 3.3.  Figure 8 shows drug particle pathline samples when the oxygen flow rate is 6 L/min and respiratory rate is 30 time/min for after 2.5 s (Figure 8(a)) and 3.5 s (Figure 8(b)) from starting time. Since there are numerous particles, in this figure, we only showed 25,900 particle pathline results, from the total of 14,400 particles. The line colors represent each particle's pathline. The particle colors show time when inside the SVJN (particle residence time). From Figures 8(a) and 8(b), it is clear that drug particles appear and float inside the SVJN's reservoir before moving through the corrugated tube and entering the mask, consistent to the velocity vector shown in Figure 7. The small figure on the right side of Figure 8(a) is an enlarged version for clearer viewing. Notice that drug particles are inhaled into the nostrils without leaking outside through the opening mask's hole due to inhaling. Oppositely, in Figure 8(b), drug particles leak out the opening mask's hole due to exhaling. These confirm that simulated pathlines are consistent with realistic breathing patterns of small children.

**Figure 9:**
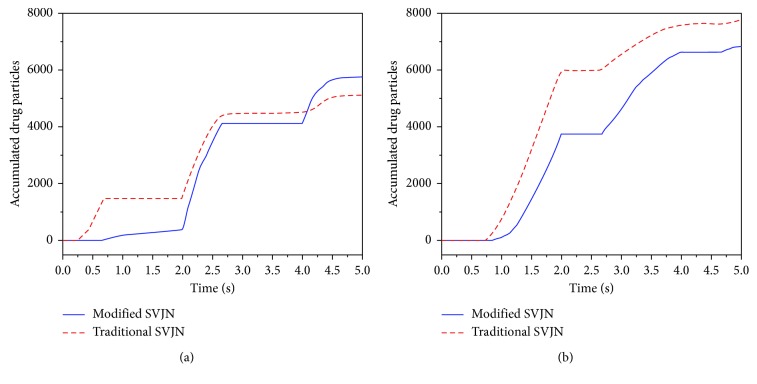
Accumulated drug particles (a) flowing into the nostrils and (b) flowing outside the opening mask's hole, for an oxygen flow rate of 6 L/min and respiratory rate of 30 time/min.

Following is the efficiency comparison between traditional and modified SVJNs. We compared by releasing 14,400 drug particles into both models with an oxygen flow rate of 6 L/min and respiratory rate of 30 time/min according to conditions mentioned in Section 3.3.  Figure 9 shows the number of drug particles that flow 9(a) into the nostrils and 9(b) outside the opening mask's hole. The vertical axis represents accumulated particles, and the horizontal axis represents the time. Because 1 in 3 of a child's breathing is inhaling while 2 in 3 is exhaling, the respiratory rate 30 time/min during the first 5 seconds in 0–0.67 s, 2.00–2.67 s, and 4.00–4.67 s are inhales and the rest are exhales. In Figure 9(a), although from 0–0.3 s is the inhaling period, no drug particles enter the nose because they have not reached the mask yet. During 2.00–2.67 s, the amount of drug particles entering the nose increases quickly because a great number of drugs have reached the mask and can be inhaled into the nostrils. For the same reason, during 4.00–4.67, drug particles will flow into the nostrils once again. This behavior is consistent both in the traditional and modified SVJNs. To compare both models along with the particle quantity that flows into the nostrils or outside, it is observed that during the first 4 seconds, the traditional SVJN helps more drugs flow into the nostrils because the traditional one does not contain a corrugated tube, thus the distance from SVJN to mask is shorter. At that moment, drug particles are concentrated inside the mask ready to be inhaled. However, after 4 s onwards, the modified SVJN enables more drug particles to enter the nostrils because at that moment, the particles are already concentrated at the mask. Importantly, since aerosol therapy requires over 5 minutes of medication, when considering the simulation results in Figure 9(a) for a period of over 5 s, the modified SVJN clearly provides patients with a larger dosage of drugs than the traditional SVJN.  Figure 9(b) shows drug particle quantities that flow through the opening mask's hole. Notice that in both models, the drug particle flows faster during 0.67–2.00 s, 2.67–4.00 s, and 4.67–5.00 s as this is the exhaling phase. Still, at all periods, drug particles in the traditional SVJN flow out more due to the lack of a corrugated tube to help slow down the drug particles. This lack also shortens the distance from SVJN to mask and lessens drug storage once compared to the modified SVJN. Most drug particles flow outside the mask instead of treating the patient. When considering the amount of drug particles flowing into the nostrils, we confidently believe that the modified SVJN is more efficient than the traditional SJN.

**Figure 10:**
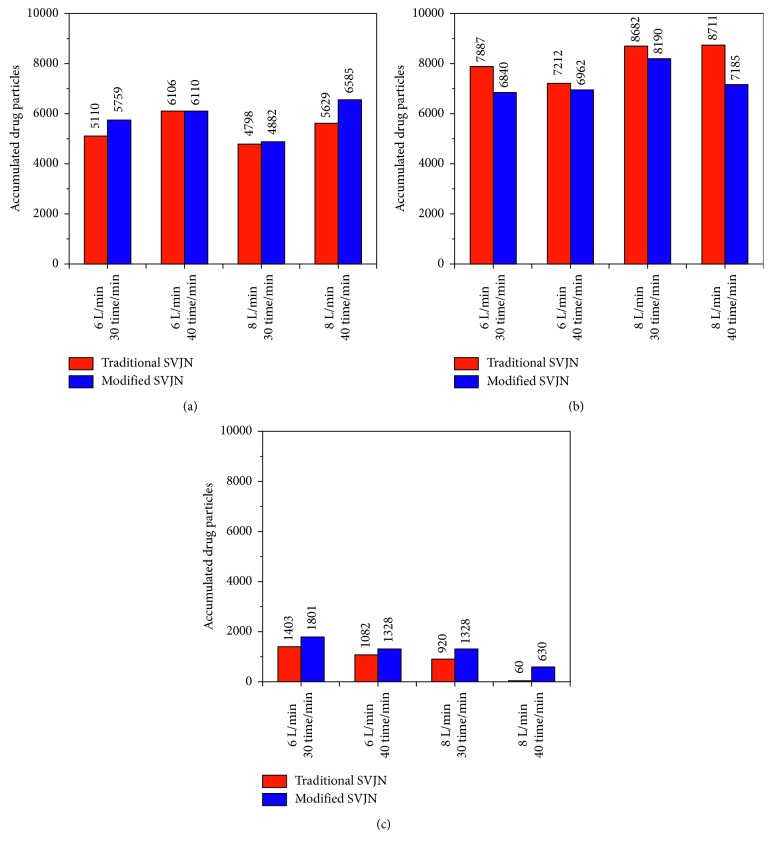
Accumulated drug particles (a) flowing into nostrils, (b) flowing outside the opening mask's hole, and (c) remaining in the SVJNs for the conditions in Table 1.

For more confidence, we simulated this experiment using 8 conditions as in Table 1. Every condition was simulated 5 times before finding an average figure. We found that the standard deviation of every information was lessen than 2.31%. All 5-time results were near to the average, had little deviations, and are statistically creditable. We used a computer to record the particle amounts that flow into the nostrils, outside the mask, and remain in the device. Simulation results are shown in Figure 10. Vertical axis represents the accumulated drug particle quantity which the computer recorded while the horizontal axis represents the simulation conditions: the oxygen flow rate and respiratory rate. From Figure 10(a), the modified SVJN helps more drug particles to flow into the nostrils than the traditional SVJN in every condition, especially when the oxygen flow rate is 8 L/min and respiratory rate is 40 time/min. More than 956 drug particles could enter the respiratory system, or 6.64% of the total amount. In Figure 10(b), though all conditions give the same results, more drug particles are lost outside by the traditional SVJN than the modified SVJN. Evidently, when the oxygen flow rate is 8 L/min and respiratory rate is 40 time/min, the traditional SVJN causes a higher drug particle lost to surroundings than modified SVJN by 1,526 particles (10.60%). From studying, we learned that 93–99% of drug particles are lost when a patient exhales [12]. From this reason, we anticipate that the modified SVJN will help decrease this loss by at least 10.60%. In Figure 10(c), all conditions gave consistent results; when the 5th second passed, most drug particles have flown out through the device with only little remaining inside the device. More particles remain in the modified SVJN than the traditional SVJN. Similar to when the oxygen flow rate is 8 L/min and the respiratory rate is 40 time/min, 570 more particles (3.96%) remain inside the modified SVJN than the traditional SVJN. More remaining particles mean that if the breathing period is lengthened, the drugs have a greater chance to flow into the body.

From all CFD results, we are confident that the modified SVJN is more effective than the traditional SVJN. As anticipated, adding a corrugated tube helps slow drug particle velocities, increase drug dispersion, enhance the dosage entering the respiratory system, decrease drug loss outside the mask, and add storage for drug particles. We applied all the information here to modify a better SVJN model that can be clinically used on children. Details will be discussed in the following section.

### 5.2. Clinical Outcomes

Results from 3 clinical studies are as follows: firstly, to investigate the effectiveness of the modified SVJN on how small children accept this redesigned equipment, by using the Mann–Whitney *U* test, the result revealed that the experimental group showed more acceptance behavior towards aerosol treatment with modified SVJN than the traditional one with statistically significant values (*p* < 0.05) (as showed in Table 3) because the additional 6-inch corrugated tube made the aerosol production less scarily noise and more friendly for small children than the traditional one. When small children accepted were satisfied with the treatment, they should stop crying, breath slowly, and synchronize with the aerosol produced.

Secondly, to investigate how the equipments work by measuring 2 physical attributes (dead volume-volume that remain in the equipment after each treatment and duration of aerosol treatment), by using ANCOVA, the results showed that 2 physical attributes which were dead volume and duration of aerosol treatment between the 2 equipment after treatment were not statistically different (as shown in Tables 4 and 5), which means that the additional 6-inch corrugated tube did not make the aerosol time longer so as the amount of dead volume compared with the one that did not have additional tube. The modified SVJN could be used as the traditional SVJN in clinical practice.

Thirdly, to investigate how it could promote the bronchodilator effect by measuring 4 clinical outcomes (oxygen saturation, respiratory rate, heart rate, and degree of wheezing) compared with the traditional one, by using the Mann–Whitney *U* test to analyze mean score of change among the 3 clinical outcomes which were oxygen saturation, respiratory rate, and degree of wheezing and ANCOVA to analyze the difference of change in heart rate, the results revealed that the experimental group obtained a higher mean score of change in oxygen saturation and degree of wheezing and greater reduction in respiratory rate per minute than control group with statistical difference (*p* < 0.05) which accepted the hypothesis (as shown in Table 6). However, the difference of change in heart rate showed no significant difference (*p* > 0.05) (as shown in Table 7). The results showed that the modified SVJN could promote higher bronchodilator effects from allowing more drug penetration to the lower lung than traditional SVJN. Nevertheless, heart rate was not found to be different which was due to the action of the bronchodilator that made heart rate increased in both groups. Longer times to evaluate the clinical outcomes were needed to explore the better outcomes.

## 6. Conclusion

In this article, we have proposed a CFD method in the transient state to modify the SVJN device. The purpose is to resolve aerosol therapy problems in treating 1–5 year-old asthmatic patients caused by loss of drugs during treatment or rejecting reactions a child may have to therapy. To solve that problem, we modified the traditional SVJN by adding 6 inches by 22 mm diameter corrugated tube. CFD simulation results for an oxygen flow rate of 6 and 8 L/min and respiratory rate of 30 and 40 time/min gave the same results; the added corrugate tube helps slow drug particle velocities up to 31.48% and increase drug dispersion and storage as well. While inhaling, the modification helps increase the drug particles entering the nostrils by 6.64%. Oppositely, while exhaling, the modified version helps lessen loss of drug particles outside the mask up to 10.60%. 3.96% drug particles remain in the modified SVJN. The corrugated tube itself acted like a reservoir that could reserve around 80 ml of the volume waiting for the small children to breath in which the hypothesis was accepted. The modified SVJN was tested for the effectiveness on how it could promote the bronchodilator drug penetrating into the lower lung compared with the traditional one by 3 clinical research studies on 1–5-year-old asthmatic children. The results from the 3 clinical studies revealed that experimental group reported higher bronchodilator effects which was shown by obtaining higher mean score of change in oxygen saturation and degree of wheezing and greater reduction in respiratory rate per minute than the control group with statistical difference (*p* < 0.05). Meanwhile, the heart rate and physical attributes (dead volume and duration of aerosol treatment) were indifferent. Other than this, small children showed more acceptance behavior towards aerosol treatment with modified SVJN than traditional one with a statistically significant value (*p* < 0.05). The use of modified small-volume jet nebulizer might be the good choice for aerosol treatment in small children because it could reserve and slow down the speed of aerosol production to make them well spread all over the reservoir and ready for small children to breath in order to get good clinical outcomes with the same physical attributes as the original device.

## Figures and Tables

**Table 1:** Conditions for CFD simulation.

Type	Oxygen flow rate (L/min)	Respiratory rate (time/min)
Traditional SVJN	6 and 8	30 and 40
Modified SVJN	6 and 8	30 and 40

**Table 2:** Oxygen velocity at positions A, B, and C obtained from measurement and simulation from the traditional and modified SVJNs with 8 L/min of oxygen flow rate.

Position/Type	Oxygen velocity (m/s)							
	Traditional SVJN				Modified SVJN			
	Simulation	Measurement	SD	Error (%)	Simulation	Measurement	SD	Error (%)
A	5.30	5.34	0.18	0.75	5.30	5.32	0.17	0.38
B	2.22	2.40	0.19	7.50	2.20	2.41	0.16	8.71
C	0.54	0.59	0.05	6.78	0.37	0.39	0.03	5.13

**Table 3:** Compared median score of change in acceptance behavior between experiment and control groups (*N*=40).

Clinical outcome	Experimental group (*n*=20)			Control group (*n*=20)			*Z*	*p*
	Min.	Max.	Median	Min.	Max.	Median		
Acceptance behavior	0	8	8	0	8	5	−1.99^*∗*^	0.034

^*∗*^ *p* < 0.05.

**Table 4:** Compared mean score of change in dead volume between experiment and control groups when controlling the effect of age (*N*=64).

Source of variation	Sum of squares	df	Mean square	*F*	*p*
Age	1.633	1	1.633	12.956	0.001
Experiment group	0.037	1	0.037	0.291	0.592
Variation	7.688	61	0.126		
Total	9.964	63			

**Table 5:** Compared mean score of change in duration of aerosol treatment between experiment and control groups when controlling the effect of age (*N*=64).

Source of variation	Sum of squares	df	Mean square	*F*	*p*
Age	7.548	1	7.548	1.597	0.211
Experiment group	2.621	1	2.621	0.555	0.459
Variation	283.604	60	4.727		
Total	295.966	63			

**Table 6:** Compared mean score of change in oxygen saturation, respiratory rate, and degree of wheezing between experiment and control groups (*N*=134).

Clinical outcome	Experimental group (*n*=68)		Control group (*n*=66)		*Z*	*p*
	Mean rank	Median	Mean rank	Median		
Oxygen saturation	73.26	2.00	61.57	1.00	−1.78^*∗*^	0.037
Respiratory rate per minute	61.22	−4.00	73.97	−2.00	−1.92^*∗*^	0.027
Degree of wheezing	62.30	−1.00	72.86	−1.00	−2.11^*∗*^	0.018

^*∗*^ *p* < 0.05.

**Table 7:** Compared mean score of change in heart rate between experimental and control groups by using ANCOVA by controlling heart rate before and after aerosol treatment (*N*=134).

Source of variance	SS	df	MS	*F*	*p*
Method of deliver aerosolized	22.87	1	22.87	110^ns^	0.741
Heart rate per minute before treatment	4262.86	1	4262.86	20.47^*∗*^	0.000
Error	27281.37	131	208.26		
Total	32029.31	133			

ns = not significant.

## Data Availability

The additional figures and animations used to support the findings of this research are included within the supplementary information files.

## Nomenclature

CFD: Computational fluid dynamics
SVJN: Small-volume jet nebulizer
*d*_*p*_: Diameter of particle (m)
*F*_*s*_: External force (N)
*k*: Turbulence kinetic energy (m^2^/s^2^)
*t*: Time (s)
*u*_*p*_: Velocity of particle (m/s)
*ρ*_*p*_: Density of particle (kg/m^3^)
*ν*: Viscosity of fluid (Pa.s)
DPM: Discrete phase model
*F*_*D*_: Drag force (N)
*F*_*s*_: Other forces acting on the particle (N)
*ε*: Dissipation rate of the eddies (m^2^/s^3^)
*g*: Gravity (m/s^2^)
*P*: Pressure (Pa)
*u*_*f*_: Velocity of fluid (m/s)
*ρ*_*f*_: Density of fluid (kg/m^3^).

## Supplementary Materials

Supplementary Materials (1) Additional Figures.pdf: The additional figures file reports the simulation process of ANSYS Fluent such as making solid and mesh models of the traditional and modified SVJN processes, GUI of the program and some boundary settings process. The details of the processes have already been included in the manuscript. (2) Animation 8L 30 times.mp4: This file shows the example of animation of SVJN for exhaling and inhaling phases in transient state for oxygen flow rate of 8L/min and respiratory rate of 30 time/sec. Reader can see the direction of drug particles in exhaling and inhaling phases for the first 5 seconds. (3) AnimationZoom 8L 30time.mp4: To support an analysis of Animation 8L 30 times.mp4:, this file shows the animation which zooms inside the modified SVJN especially near the nostrils areas. Arrow up means the inhaling phase while arrow down means the exhaling phase. Color of drug particles is velocity. We recorded the number of drug particles in both phases versus time to analyse the efficacy of the SVJN. Both animation files support the discussion and analysis of Figures 8-10.

## References

[1] Khilnani G. C., Banga A. (2008). Aerosol therapy. *Indian Journal of Chest Diseases and Applied Sciences*.

[2] Schuepp K. G., Devadason S., Roller C., Wildhaber J. H. (2004). A complementary combination of delivery device and drug formulation therapy in preschool children. *Swiss Medical Weekly*.

[3] Ahren R. C. (2005). The role of the MDI and DPI in pediatric patient: children are not just miniature adults. *Respiratory care*.

[4] Amirav I., Balanov I., Gorenberg M., Groshar D., Lunder A. S. (2003). Nebuliser hood compared to mask in wheezy infants: aerosol therapy without tears!. *Archives of Disease in Childhood*.

[5] Amirav I., Oron A., Tal G. (2005). Aerosol delivery in respiratory syncytial virus bronchiolitis: hood or Face Mask?. *Journal of Pediatrics*.

[6] Inthavong K., Ge Q., Se C. M. K., Yang W., Tu J. Y. (2011). Simulation of sprayed particle deposition in a human nasal cavity including a nasal spray device. *Journal of Aerosol Science*.

[7] Thongsri J., Pimsarn M. (2015). Optimum airflow to reduce particle contamination inside welding automation machine of hard disk drive production line. *International Journal of Precision Engineering and Manufacturing*.

[8] Khaokom A., Thongsri J. (2017). Feasibility study for installing machine in production line to avoid particle contamination based on CFD simulation. *IOP Conference Series: Materials Science and Engineering*.

[9] Thongsri J. (2017). A problem of particulate contamination in an automated assembly machine successfully solved by CFD and simple experiments. *Mathematical Problems in Engineering*.

[10] Kaewbumrung M., Orankitjaroen S., Boonkrong P., Nuntadilok B., Wiwatanapataphee B. (2018). Numerical simulation of dispersed particle-blood flow in the stenosed coronary arteries. *International Journal of Differential Equations*.

[11] Zhou Y., Lee C., Wang J. (2018). The computational fluid dynamics analyses on hemodynamic characteristics in stenosed arterial models. *Journal of Healthcare Engineering*.

[12] O’Callaghan C., Barry P. W. (1997). The science of nebulized drug delivery. *Thorax*.

[13] O’Callaghan C. (1997). Delivery systems: the science. *Pediatric Pulmonology*.

[14] Ari A. (2014). Jet, ultrasonic, and mesh nebulizers: an evaluation of nebulizers for better clinical outcomes. *Eurasian Journal of Pulmonology*.

[15] Turbulence, Fluent Theory Guide 17.1, Chapter 4, 2016

[16] Menter F. R. Zonal two equation *k*-*ω* turbulence models for aerodynamic flows.

[17] Multiphase Flow, Fluent Theory Guide 17.1, Chapter 17, 2016

[18] Discrete Phase, Fluent Theory Guide 17.1, Chapter 16, 2016

[19] Lelong N., Vecellio L., de Gélicourt Y. S., Tanguy C., Diot P., Junqua-Moullet A. (2013). Comparison of numerical simulations to experiments for atomization in a jet nebulizer. *PLoS One*.

