# Supplementary material for: Modified Small-Volume Jet Nebulizer Based on CFD Simulation and Its Clinical Outcomes in Small Asthmatic Children
Source: J Healthc Eng. 2019 Jun 10;2019:2524583. doi: 10.1155/2019/2524583 (PMC6590536; doi:10.1155/2019/2524583)

Additional Figures for Review Process

Modified SVJN

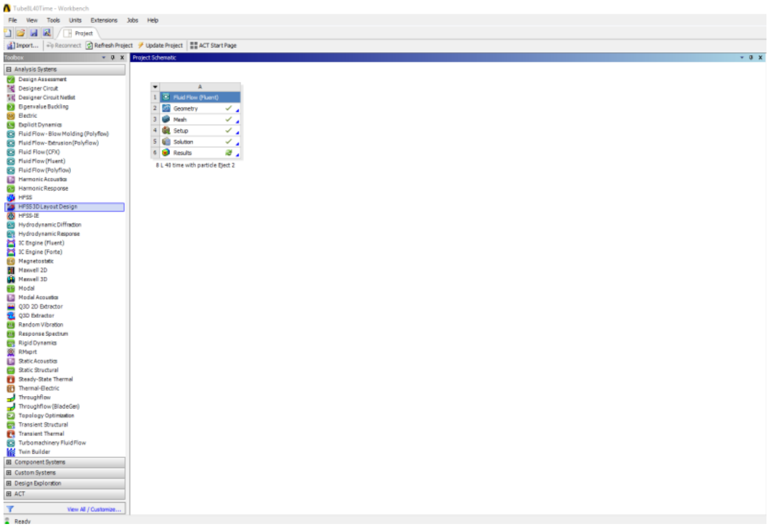

Workbench in ANSYS Program for the modified SVJN

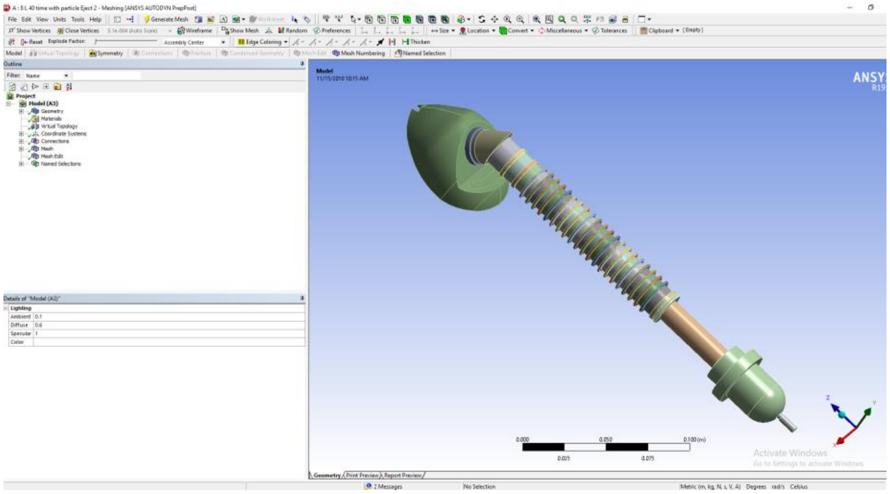

Solid Model of modified SVJN

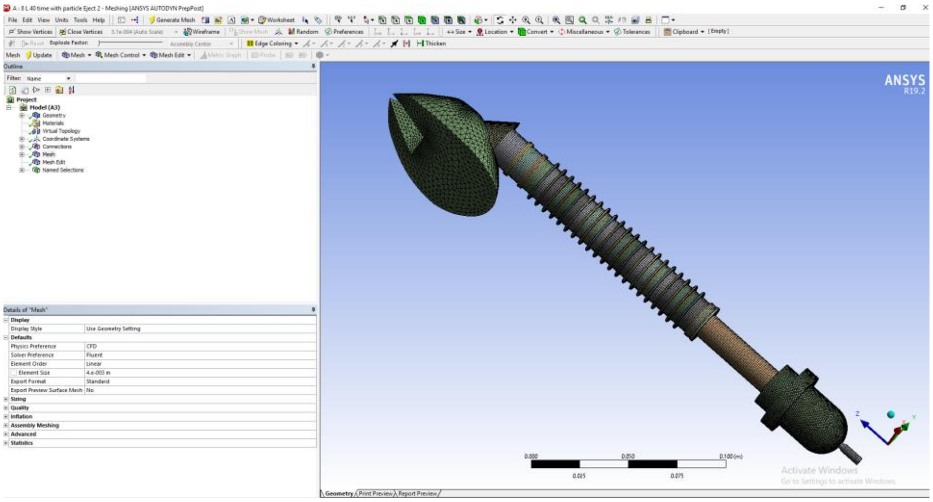

Mesh Model of modified SVJN

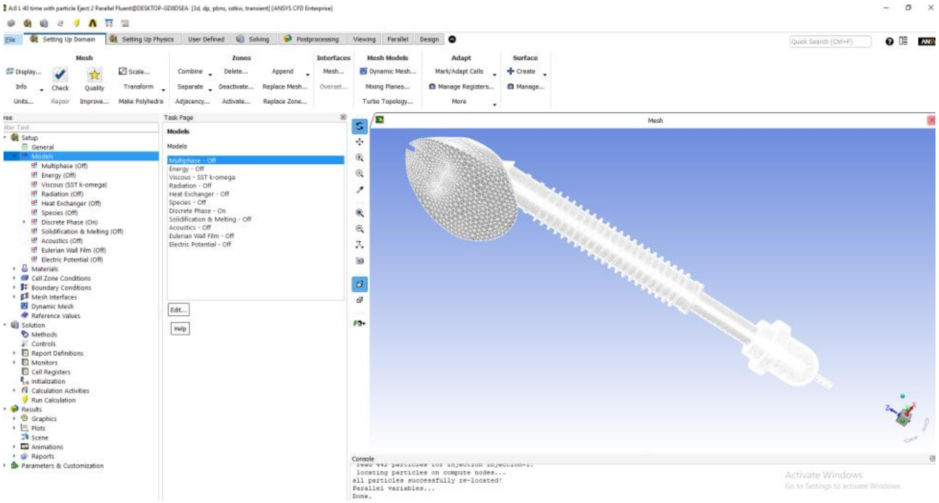

Graphic User Interface of ANSYS Fluent

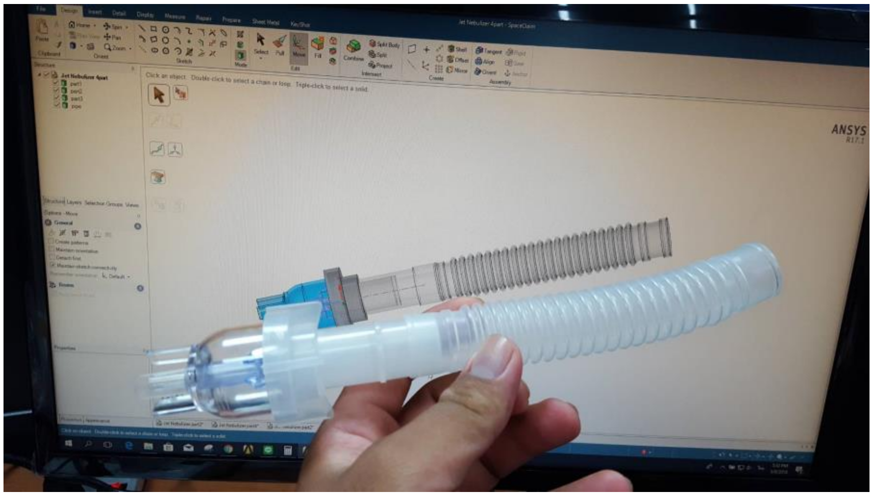

Comparison between the actual model of modified SVJN and CAD model

Traditional SVJN

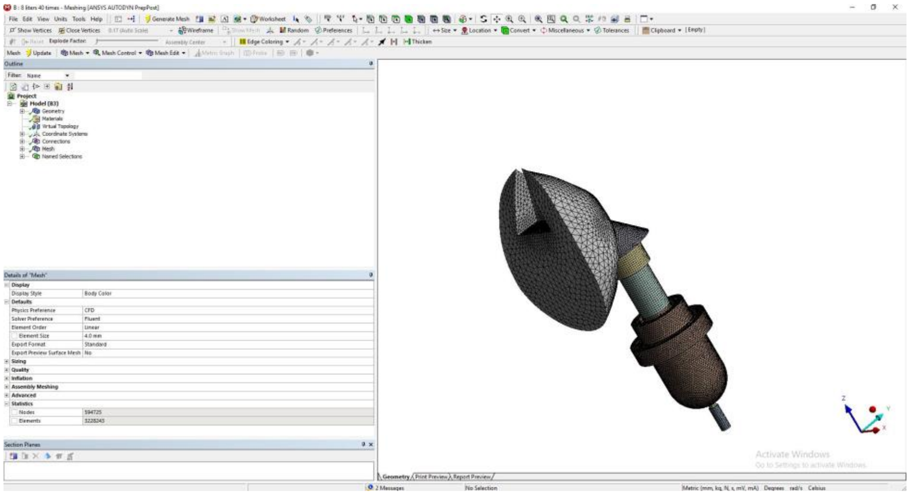

Mesh model of the traditional SVJN

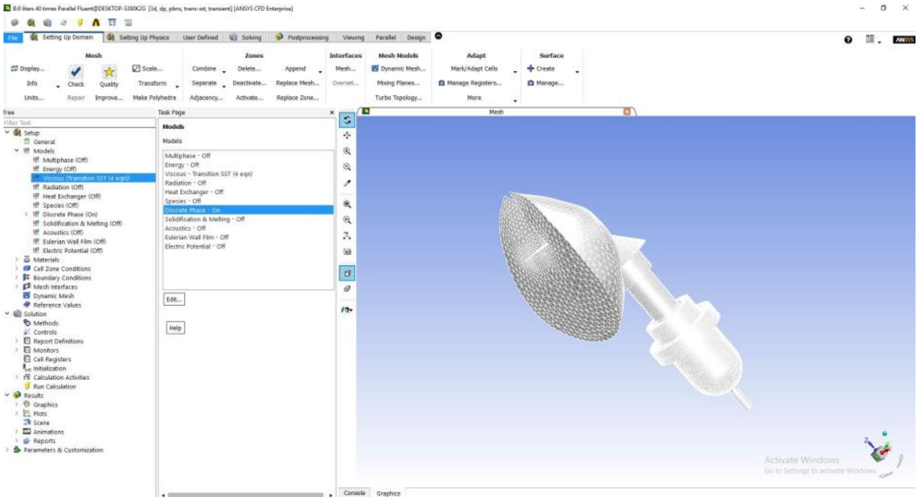

Graphic user interface of ANSYS Fluent for traditional SVJN

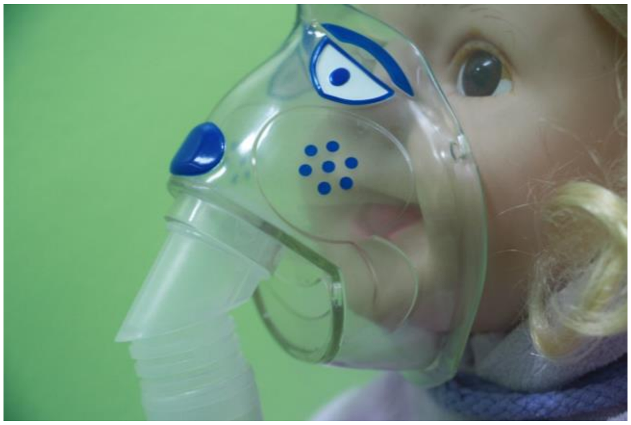

Sample picture of mask and conlugated tube, drug mist inside the SVJN is very difficult to view so the CFD is the best way to understand its behavior.

Samples of animations have been included in the files.

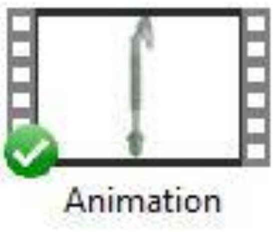

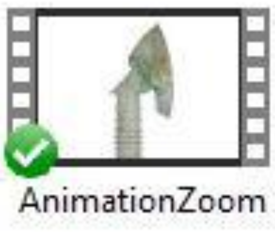

Supplement: Supplementary Materials — (1) Additional Figures.pdf: The additional figures file reports the simulation process of ANSYS Fluent such as making solid and mesh models of the traditional and modified SVJN processes, GUI of the program and some boundary settings process. The details of the processes have already been included in the manuscript. (2) Animation 8L 30 times.mp4: This file shows the example of animation of SVJN for exhaling and inhaling phases in transient state for oxygen flow rate of 8L/min and respiratory rate of 30 time/sec. Reader can see the direction of drug particles in exhaling and inhaling phases for the first 5 seconds. (3) AnimationZoom 8L 30time.mp4: To support an analysis of Animation 8L 30 times.mp4:, this file shows the animation which zooms inside the modified SVJN especially near the nostrils areas. Arrow up means the inhaling phase while arrow down means the exhaling phase. Color of drug particles is velocity. We recorded the number of drug particles in both phases versus time to analyse the efficacy of the SVJN. Both animation files support the discussion and analysis of Figures 8-10. [file 2524583.f1.zip › Additional Figures.pdf]
